# Study on the Dynamic Recrystallization Behavior of 47Zr-45Ti-5Al-3V Alloy by CA–FE Simulation

**DOI:** 10.3390/ma14102562

**Published:** 2021-05-14

**Authors:** Wenwei Zhang, Qiuyue Yang, Yuanbiao Tan, Ya Yang, Song Xiang, Fei Zhao

**Affiliations:** Guizhou Key Laboratory of Materials Mechanical Behavior and Microstructure, College of Materials and Metallurgy, Guizhou University, Guiyang 550025, China; Zww19960916@163.com (W.Z.); yangqy979@126.com (Q.Y.); sxiang@gzu.edu.cn (S.X.); fzhao@gzu.edu.cn (F.Z.)

**Keywords:** 47Zr-45Ti-5Al-3V alloy, hot-working, DRX behavior, FEM, CA

## Abstract

The dynamic recrystallization (DRX) behavior of 47Zr-45Ti-5Al-3V alloy was studied by using the experiment and numerical simulation method based on DEFORM-3D software and cellular automata (CA) over a range of deformation temperatures (850 to 1050 °C) and strain rates (10^−3^ to 10^0^ s^−1^). The results reveal that the DRX behavior of 47Zr-45Ti-5Al-3V alloy strongly depends on hot-working parameters. With rising deformation temperature (*T*) and decreasing strain rate ($\dot{\varepsilon }$), the grain size ($d_{DRX}$) and volume fraction ($X_{DRX}$) of DRX dramatically boost. The kinetics models of the $d_{DRX}$ and $X_{DRX}$ of DRX grains were established. According to the developed kinetics models for DRX of 47Zr-45Ti-5Al-3V alloy, the distributions of the $d_{DRX}$ and $X_{DRX}$ for DRX grains were predicted by DEFORM-3D. DRX microstructure evolution is simulated by CA. The correlation of the kinetics model is verified by comparing the $d_{DRX}$ and $X_{DRX}$ between the experimental and finite element simulation (FEM) results. The nucleation and growth of dynamic recrystallization grains in 47Zr-45Ti-5Al-3V alloy during hot-working can be simulated accurately by CA simulation, comparing with FEM.

## 1. Introduction

Zirconium alloys owns low thermal neutron absorption cross-section, superior mechanical properties for long term operations in high pressure and adequate corrosion resistance in contact with high-temperature water, which plays a significant role in structural materials of the aerospace field [1,2,3]. With the rapid development of the aviation industry, the increasing requirement in the mechanical properties was needed for the structural materials of the aerospace field. Recently, a series of new ZrTiAlV alloys with an ultrahigh strength were designed [4,5,6,7,8,9]. The mechanical properties of key components were depended on the microstructure produced during hot-working of the ZrTiAlV alloys, which was effected by hot processing parameters. Therefore, it is essential to deeply reveal the microstructure evolution and deformation mechanism of ZrTiAlV alloys at various hot-processing conditions. In the previous work, the hot-deformation behavior of ZrTiAlV alloys was investigated, and the constitutive equation and processing maps have established [7,8,9,10,11]. The hot-deformation behavior of new 47Zr-45Ti-5Al-3V alloy is significantly different from that of traditional zirconium alloys [12,13,14,15]. For the 47Zr-45Ti-5Al-3V alloy, the dynamic recrystallization is not easily occurred at low deformation temperature and high strain rate. Generally, DRX is regarded as an excellent way to refine the grain size of metals during the hot-working. However, no published investigation on the DRX kinetics behavior of 47Zr-45Ti-5Al-3V alloys has been carried out. Thus, it is critical to understand the microstructure evolution in the process of DRX and construct the DRX kinetics model for improving the mechanical properties of 47Zr-45Ti-5Al-3V alloy.

Recently, with the rapid increase in computer performance, the DRX kinetics of metals during hot-working has been qualitatively studied by using the software of FEM and CA [16,17,18,19,20,21,22,23,24]. Irani et al. [16] investigated the DRX kinetics of the AA6060 aluminum alloy by the FEM method. The results demonstrated that controlling the number of element points can effectively improve efficiency and increase the accuracy of FEM. Ji et al. [17] embed the DRX kinetics model for 33Cr23Ni8Mn3N alloy into the DEFORM-3D software to characterize the relationship between hot-working parameters and the $d_{DRX}$ and $X_{DRX}$ of DRX grains. The result manifests a good consistent between the simulation and experimental results. Wu et al. [18] utilized a cellular automaton (CA) coupled with FEM by means of ABAQUS software to study the DRX microstructure evolution of AZ61 alloy. The simulation results correspond well to the experimental results. NithinBaler et al. [19] revealed the DRX mechanism of *γ*′-L12 alloy by the FEM methods, which was discontinuous dynamic recrystallization (DDRX). Geng et al. [20] also reported that the DRX evolution of GH4169 superalloy during hot-working can be better predicted by FEM software integrated with the developed kinetics model. Zhang et al. [21] studied the microstructure evolution of 7055 aluminum alloy in the rolling process by using FEM and CA methods, and found that CA method can more accurately simulate the evolution of DRX. Li et al. [22] utilized the 3D-CA method to describe the DRX behavior and mechanical response of the titanium alloy during the uneven deformation, which showed a good consistency with the results obtained by experimental test. All in all, the CA and FEM methods have been widely regarded as valid ways to predict the microstructure evolution in the DRX process of metals and alloys.

In order to reveal the DRX behavior and construct the DRX kinetics model of 47Zr-45Ti-5Al-3V alloy, in this present work, the CA–FE method is used to study the DRX behavior of 47Zr-45Ti-5Al-3V alloy from the macro- and micro-scales. A developed kinetics model of the DRX for 47Zr-45Ti-5Al-3V alloy was established. The microstructure evolution of the alloy in the process of DRX was analyzed by DEFORM-3D software and CA integrated with the developed kinetics and dislocation models. Additionally, a comparison of the difference between the experimental and simulated results has been carried out to testify the validity of FE–CA simulation.

## 2. Experiment

**Figure 1:**
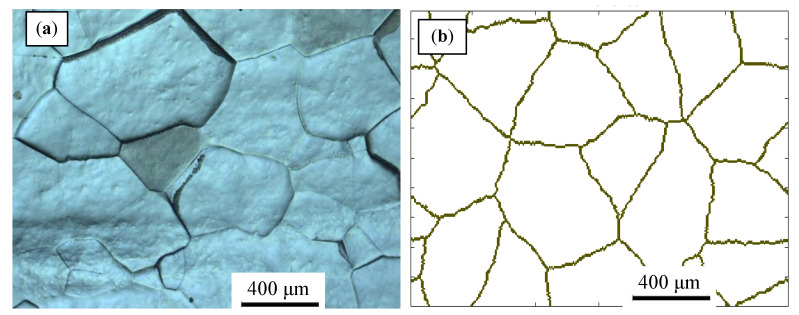
(**a**) Microstructure of the 47Zr-45Ti-5Al-3V alloy solution-treatment at 1050 °C for 0.5 h. (**b**) Initial microstructure simulated by CA.

A forged 47Zr-45Ti-5Al-3V alloy (wt.%) was employed in this work. The specific preparation process has been introduced in the previous work [8]. The phase transformation temperature of β → α + β for 47Zr-45Ti-5Al-3V alloy is 703 °C [5]. The forged alloy was solution-treatment at 1050 °C for 0.5 h and subsequently water-quench. Figure 1a depicts the initial microstructure of the solution-treated alloy with an average grain size of about 450 μm.

**Figure 2:**
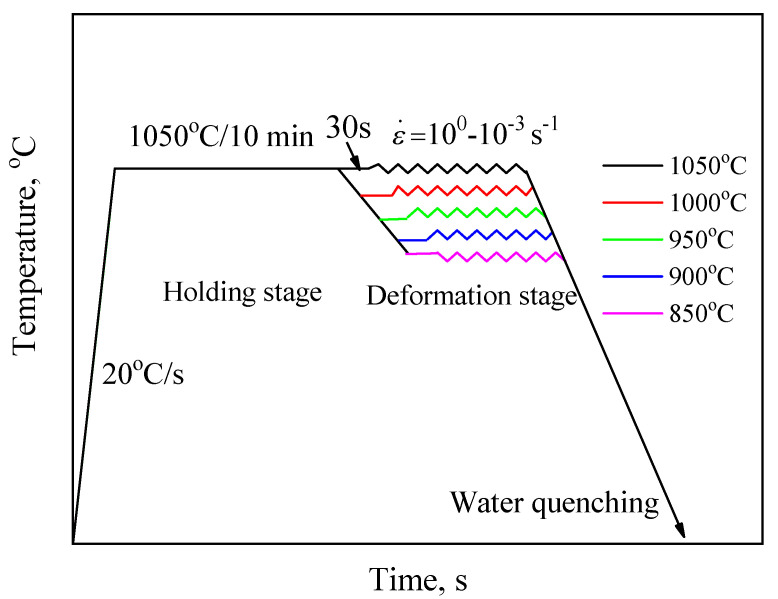
Schematic diagram of the experimental tests in this work.

Cylindrical samples of ø8 × 12 mm were prepared by the wire cutting. To analyze the DRX behavior and construct the DRX kinetics model of 47Zr-45Ti-5Al-3V alloy, hot compression test was conducted on a Gleeble 3500 at various testing conditions. The deformation temperature was set in the range of 850 to 1050 °C, and the strain rate was set in the range of 10^−3^ to 10^0^ s^−1^. The schematic diagram of the experimental tests is shown in Figure 2. The testing samples were compressed to a true strain of 0.7. For preserving the microstructure of the deformed samples, the deformed samples were rapidly water-quenched to 20 °C.

For optical microstructure observation (OM) by using Leica DMI5000M, the deformed samples were ground and then etched by chemical means. The etching solution is a Kroll reagent of 15% HF, 40% HNO_3_ and 45% H_2_O (Vol.%). The value of the $d_{DRX}$ and $X_{DRX}$ of DRX grains was counted by the Image-Pro software (Media Cybernetics, Silver Spring, MD, USA).

## 3. Results and Discussion

### 3.1. Hot-Deformed Microstructure

**Figure 3:**
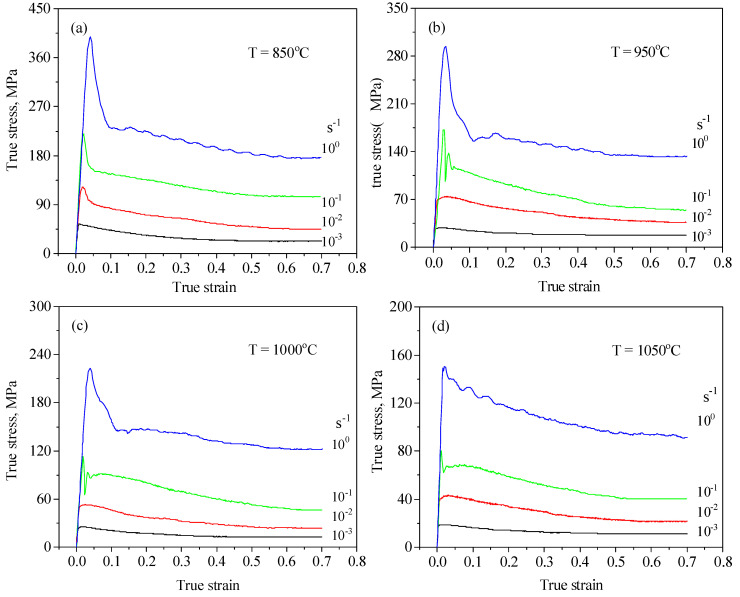
Typical stress–strain curves for the ZrTiAlV alloy at different temperatures [11]. (**a**) T = 850 °C; (**b**) T = 950 °C; (**c**) T = 1000 °C; (**d**) T = 1050 °C.

**Figure 4:**
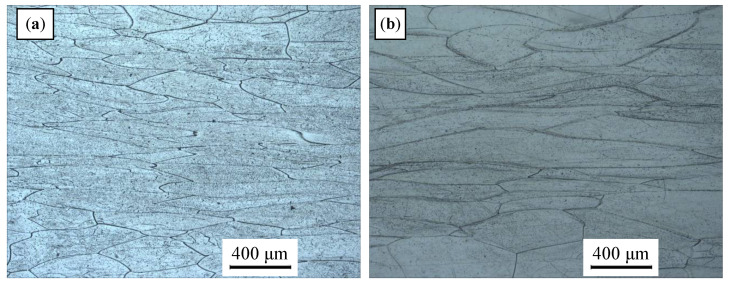

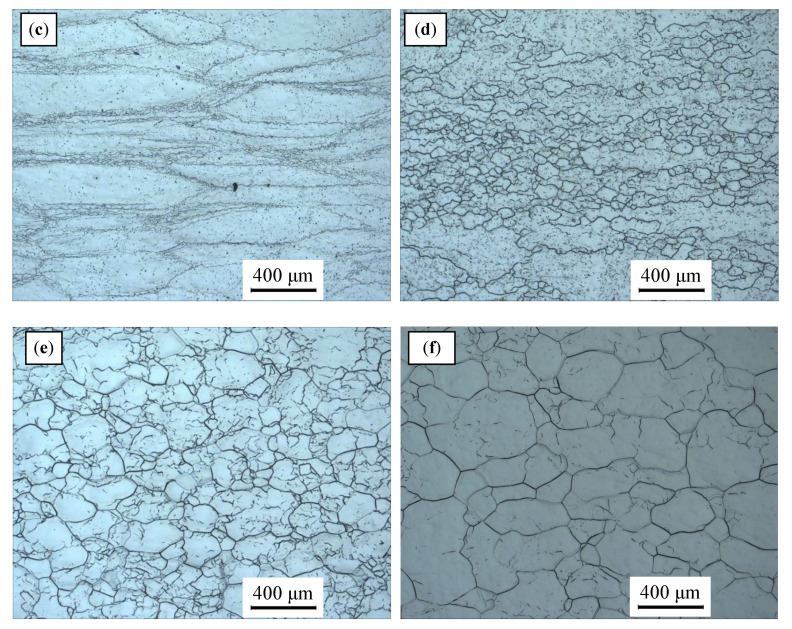
Optical microstructures of ZrTiAlV alloy under different hot-working conditions at the true strain of 0.7: (**a**) 850 °C, 1 s^−1^; (**b**) 900 °C, 1 s^−1^; (**c**) 950 °C, 0.1 s^−1^; (**d**) 1000 °C, 0.01 s^−1^; (**e**) 1000 °C, 0.001 s^−1^; and (**f**) 1050 °C, 0.001 s^−1^.

Figure 3 presents typical stress–strain curves for the ZrTiAlV alloy at different temperatures. It is seen that the stress–strain curves illustrate a dynamic recovery characteristic at a low deformation temperature and high strain rate, while showing a dynamic recrystallization characteristic at a high deformation temperature and low strain rate. Figure 4 displays the representative deformed microstructures of the 47Zr-45Ti-5Al-3V alloy. At the strain rate of 1 s^−1^, the initial grains of the samples deformed at the temperatures of 850 and 900 °C were elongated perpendicular to the compression direction of deformed samples, indicating that only DRV occurred. When the samples deformed at 950 °C/1 s^−1^, a few fine grains of DRX formed at the grain boundaries of deformed grains, showing that dynamic recrystallization firstly took place under this deformation condition. As the deformation temperature increases and the $\dot{\varepsilon }$ decreases, the grain boundary migration rate is accelerated and the $X_{DRX}$ and $d_{DRX}$ of DRX grains increase. At the hot-working parameters of 1000 °C and 0.001 s^−1^, the microstructure with uniform equiaxed grains can be achieved, revealing that a full DRX occurred during hot-working. When the *T* further raised to 1050 °C, there were obvious coarsening for the DRX grains. It is attributed to a fact that high deformation temperature and low $\dot{\varepsilon }$ provide enough energy and sufficient time for dynamic recrystallization to nucleate and grow, so the dynamic recrystallization is more sufficient under this condition.

### 3.2. Peak Strain and Critical Strain

According to the flow curves obtained from hot-working, the peak strain ($\varepsilon_{p}$) of each flow curve is as listed in Table 1. It was observed that the $\varepsilon_{p}$ gradually declined with the increment deformation temperature of at a given strain rate.

In previous work, the value of deformation activation energy (Q) was calculated to be 207.7 kJ/mol. The constitutive equation of the 47Zr-45Ti-5Al-3V alloy has been established according to the hyperbolic-sine Arrhenius-type equation as follows [11]:

(1) $$\dot{\varepsilon }=6.7\times 10^{8}{\left[\sinh(\alpha \sigma_{p})\right]}^{3.09}\exp(-\frac{207700}{RT})$$

**Figure 5:**
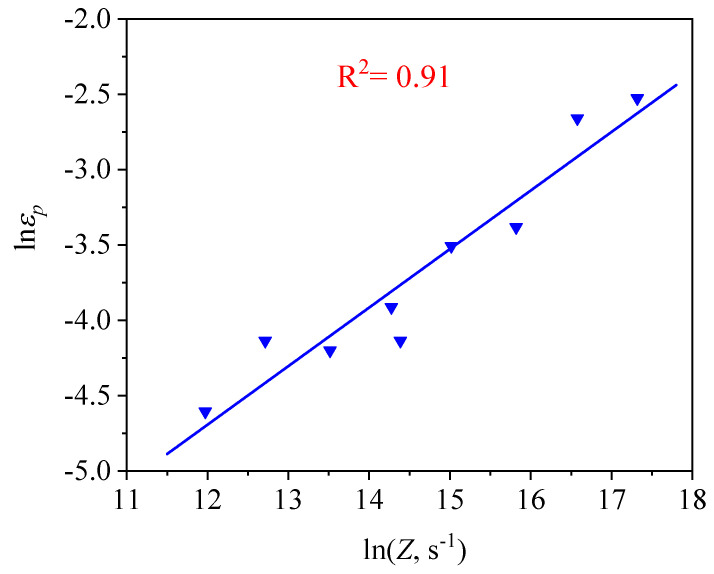
Relationship between the lnZ and ln$\varepsilon_{p}$.

Hence, the relationship between the $\varepsilon_{p}$ and Zener–Hollomon parameter ($Z=\dot{\varepsilon }\exp(\frac{Q_{}}{RT})$) can be characterized, as shown in Figure 5. The relation between the $\varepsilon_{p}$ and Z can be written as the following formula [25,26,27,28]:

(2) $$\varepsilon_{p}=8.61\times 10^{-5}Z^{0.39}$$

**Figure 6:**
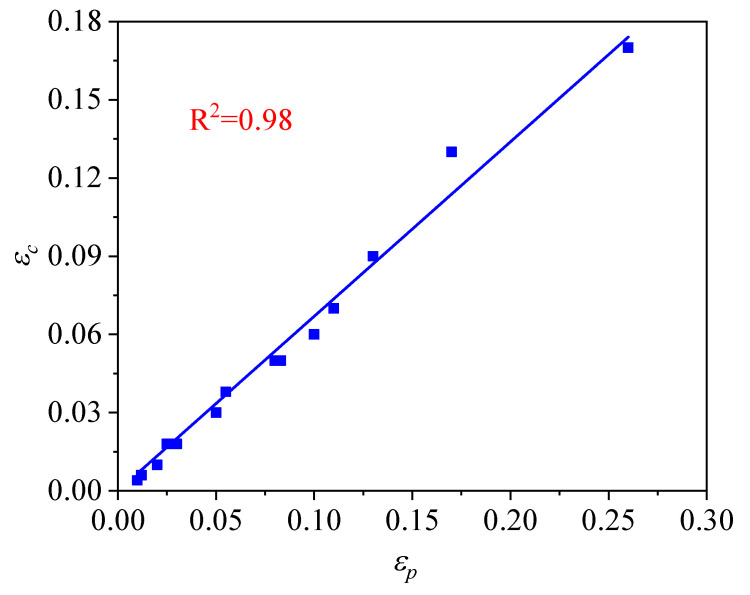
Relationship between the $\varepsilon_{c}$ and $\varepsilon_{p}$.

Generally, when the strain reaches a critical strain ($\varepsilon_{c}$) during hot-working, the DRX can occur due to the driving of dislocation accumulation and entanglement [29]. The critical strain ($\varepsilon_{c}$) is corresponding to the start point of DRX, which can be achieved by a turning point (∂(∂θ/∂σ)/∂σ=0) gained from the curve of strain hardening rate (θ) corresponding to the true stress (σ) [30,31]. In this present study, the values of $\varepsilon_{c}$ at various hot-working parameters were listed in Table 1. The relation between $\varepsilon_{c}$ and $\varepsilon_{p}$ can be expressed by the following [32]:

(3) $$\varepsilon_{c}=\beta \varepsilon_{p}$$

where the value of β (material constant) can be achieved to be 0.67 by the data in Figure 6. By integrating Equation (2) with Equation (3), the $\varepsilon_{c}$ can be written as follows:

(4) $$\varepsilon_{c}=5.77\times 10^{-5}Z^{0.39}$$

### 3.3. Kinetics Model of DRX

The DRX behavior consisted of nucleation and growth during hot-working. For the discontinuous recrystallization mechanism, the nucleus of DRX grains generally formed in the grain boundaries, and then grew toward the interior of the grains with a high density nearby the grain boundaries. The $X_{DRX}$ of DRX grains was significantly affected by the hot-working parameters, which is expressed by using the JMAK equation [32,33]:

(5) $$X_{DRX}=1-\exp\left[-k\times {\left(\frac{\varepsilon -\varepsilon_{c}}{\varepsilon_{p}}\right)}^{n}\right]$$

where k and n represent the Avrami material constants. In previous work, the measuring of the $X_{DRX}$ was usually obtained by OM and the Electron Backscatter Diffraction (EBSD) technique [33]; however, those methods require considerable calculations and are expensive to carry out. In order to solve the above problems, $X_{DRX}$ is extensively computed based on the relationship between $X_{DRX}$ and σ in the process of hot-working, which can be expressed as follows [34,35]:

(6) $$X_{DRX}=\frac{\sigma_{p}-\sigma }{\sigma_{p}-\sigma_{ss}}$$

where $\sigma_{p}$ and $\sigma_{ss}$ are on behalf of the peak stress and the steady flow stress of the flow curve, respectively. To calculate the values of k and n in Equation (5), we take the natural logarithm on both sides of Equation (5), as written in Equation (7):

(7) $$\ln[-\ln(1-X_{DRX})]=\ln k+n\ln[(\varepsilon -\varepsilon_{c})/\varepsilon_{p}]$$

**Figure 7:**
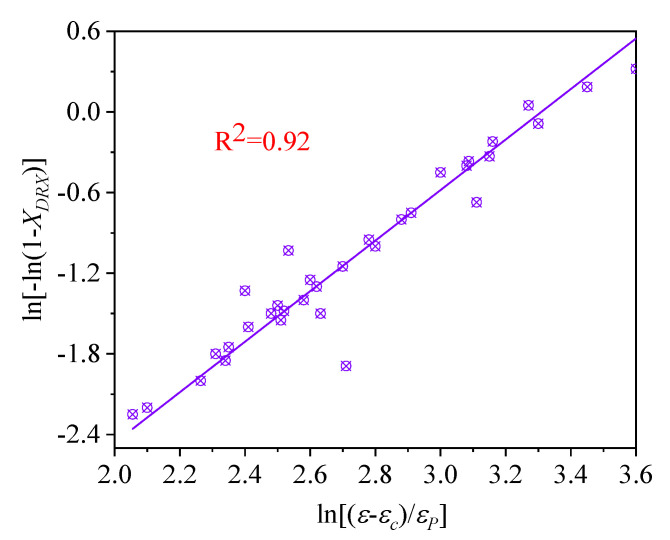
Relationship between $\ln[-\ln(1-X_{DRX})]$ and $\mathrm{In}[(\varepsilon -\varepsilon_{c})/\varepsilon_{p}]$.

The k and n (average values) can be calculated as 0.0021 and 1.88 by the data in Figure 7, respectively. Hence, the kinetic model of *DRX* was expressed as follows:

(8) $$X_{DRX}=1-\exp\left[-0.0021\times {\left(\frac{\varepsilon -\varepsilon_{c}}{\varepsilon_{p}}\right)}^{1.88}\right]$$

**Figure 8:**
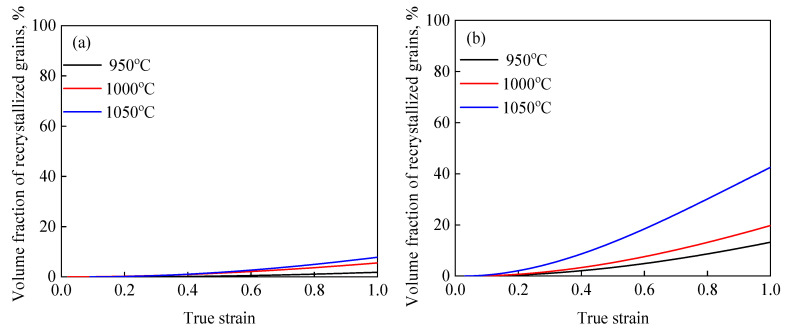

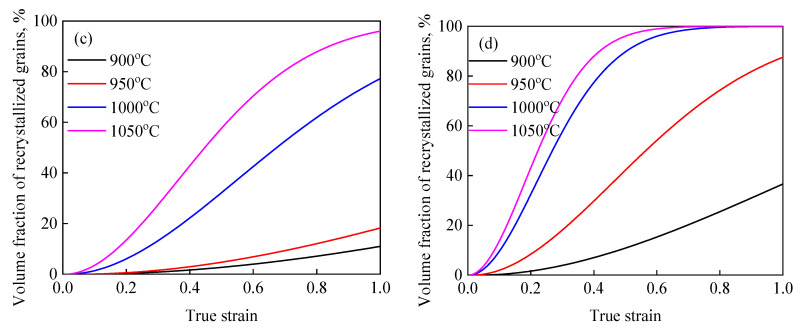
Variation in the $X_{DRX}$ with true strain at different temperatures of (**a**) 1 s^−1^, (**b**) 0.1 s^−1^, (**c**) 0.01 s^−1^ and (**d**) 0.001 s^−1^.

The $X_{DRX}$-ε curves were obtained from the DRX kinetics model of 47Zr-45Ti-5Al-3V alloy at various deformation conditions, as shown in Figure 8. So, as to further analyze the growth behavior of DRX grains, the $d_{DRX}$ as a function of *Z* parameter was characterized by the follow [35]:

(9) $$\;d_{DRX}=CZ^{m_{1}}$$

where the C and $m_{1}$ are constants. The Equation (9) can be rewritten as the form of Equation (10):

(10) $$\;\ln d_{DRX}=\ln C+m_{1}\ln Z$$

**Figure 9:**
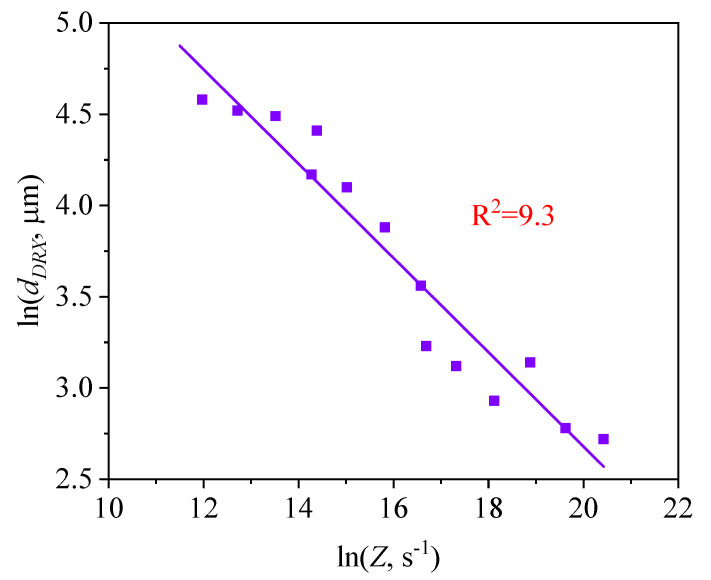
Relationship between ln$d_{DRX}$ and lnZ.

The variation on the $d_{DRX}$ with hot-working parameters was depicted in Figure 9. The material constants were calculated as C = 2565.73 and $m_{1}$ = −0.25, respectively. Therefore, the connection between the $d_{DRX}$ and the *Z* parameter can be showed as follows:

(11) $$\;d_{DRX}=2565.73Z^{-0.25}$$

### 3.4. FEM of DRX Behavior

**Figure 10:**
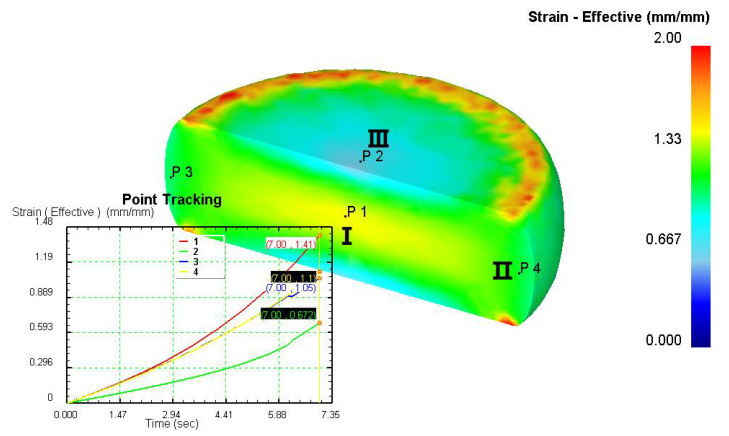
Diagram of deformation area.

Based on the DRX kinetic equation established above, the isothermal forging process was simulated by the DEFORM-3D software (Scientific Forming Technologies Corporation, Columbus, OH, USA) During the process of isothermal forging simulation, the elastic deformation can be usually ignored. Therefore, the workpiece was treated as an object with plasticity, while the tools were treated as an object with rigidity. For improving the efficiency of FEM calculation and increasing the accuracy of the simulation results, the FE model was established by symmetrical half cylinder. The sample for FEM was segmented in terms of tetrahedral meshes. The numbers of the meshes and the nodes were set to 26,538 and 4726, respectively. The top die was set as movable, while the bottom die was fixed. The coefficient of the friction was set to be 0.3 [33]. For the keeping consistency between experimental and the simulation results, the temperature of all items in the model was set to be consistent with the experimental temperature during the process of FEM. Figure 10 shows the sketch map of effective stress for FEM at the true strain of 0.7. It is seen that the deformation region is mainly divided into three different regions, according to the degree of the deformation, as marked in Figure 10. A heavy deformation took place in region *Ⅰ*, while only a slight deformation was observed in region *Ⅱ*. Usually, the region *II* is defined as the free deformation area. It is noted that non-deformation was presented in region *Ⅲ*, namely “dead zone” [33,34]. This indicates that the plastic deformation of the samples is uneven.

Table 2 displays the distribution cloud maps of the $d_{DRX}$ for 47Zr-45Ti-5Al-3V alloys. It is worth noting that the $d_{DRX}$ distribution is uneven in different regions, due to the non-uniformity of deformation in the compression process. Under low *T* and high $\dot{\varepsilon }$ conditions, only a few DRX grains with small size formed in region *Ⅰ*, while dynamic recrystallization cannot occur in region *Ⅲ*, where the size of grains keeps the original grain size. With the augment of *T* and the drop of $\dot{\varepsilon }$, the size of DRX grains gradually increased. When the deformation condition is 1050 °C/0.001 s^−1^, the size of DRX grains increased to approximately 128 μm. It is worth noting that the size of DRX grains in region *Ⅲ* is higher than that in region *Ⅱ*. Table 3 depicts the distribution cloud maps of the $X_{DRX}$ deformed at the true strain of 0.7. It can be observed from Table 3 that the $X_{DRX}$ is obviously low under low *T* and high $\dot{\varepsilon }$ conditions. With the augment of *T* and the drop of $\dot{\varepsilon }$, the $X_{DRX}$ gradually increased. It is also important to note that the distribution of the $X_{DRX}$ is also non-uniform in different regions during hot-working. Regions with a large degree of deformation have higher deformation storage energy, thereby promoting the nucleation of DRX [36,37,38,39,40]. The $X_{DRX}$ in region *Ⅰ* is higher than that in regions *Ⅱ* and *Ⅲ*.

**Figure 11:**
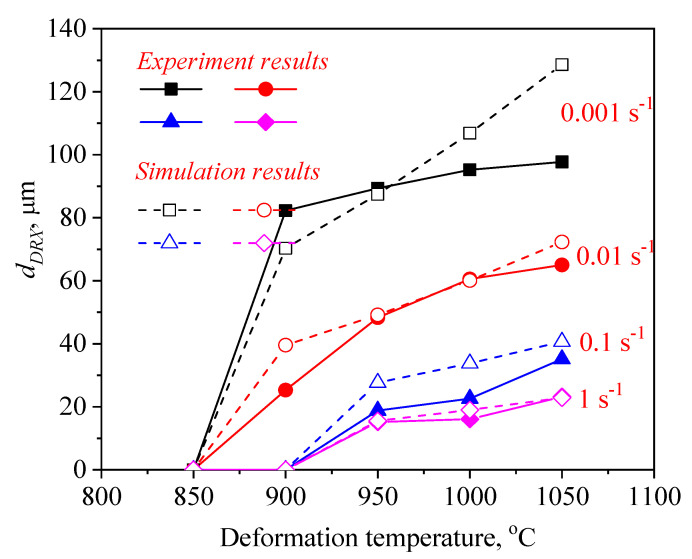
A comparison between the experimental and FE results of the grain size of recrystallized grains ($d_{DRX}$).

**Figure 12:**
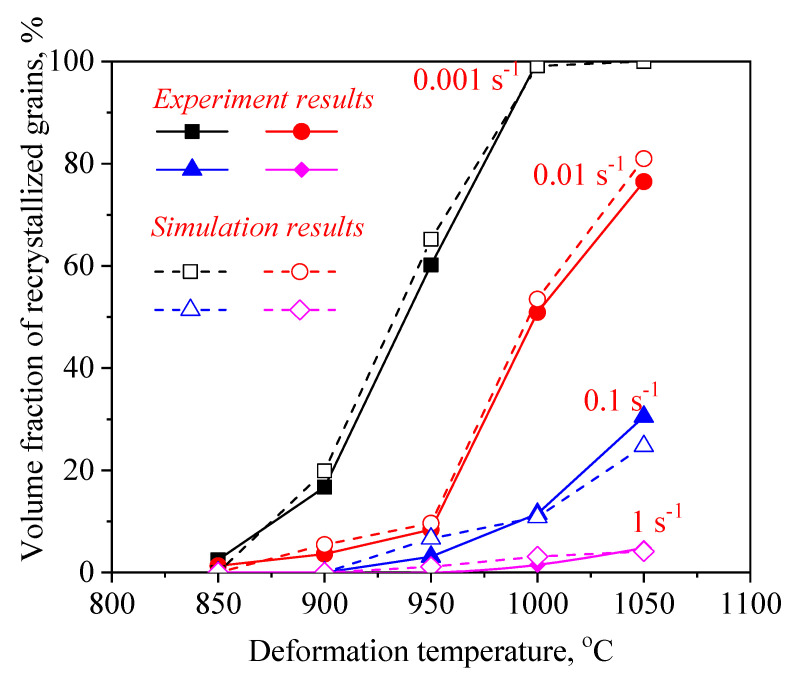
A comparison between the experimental and FE simulation results of the volume fraction of DRX ($X_{DRX}$).

**Figure 13:**
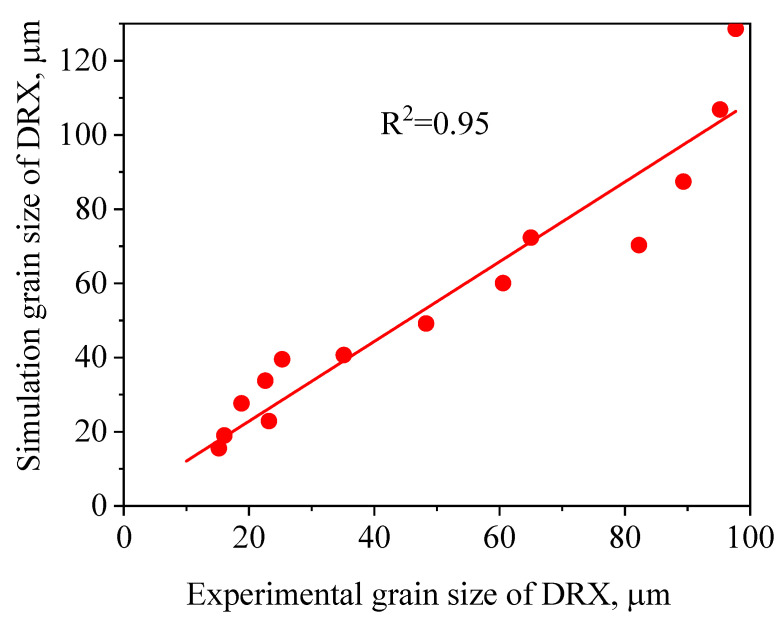
Correlation between the FEM and experimental grain size of DRX for 47Zr-45Ti-5Al-3V alloys after hot deformation.

**Figure 14:**
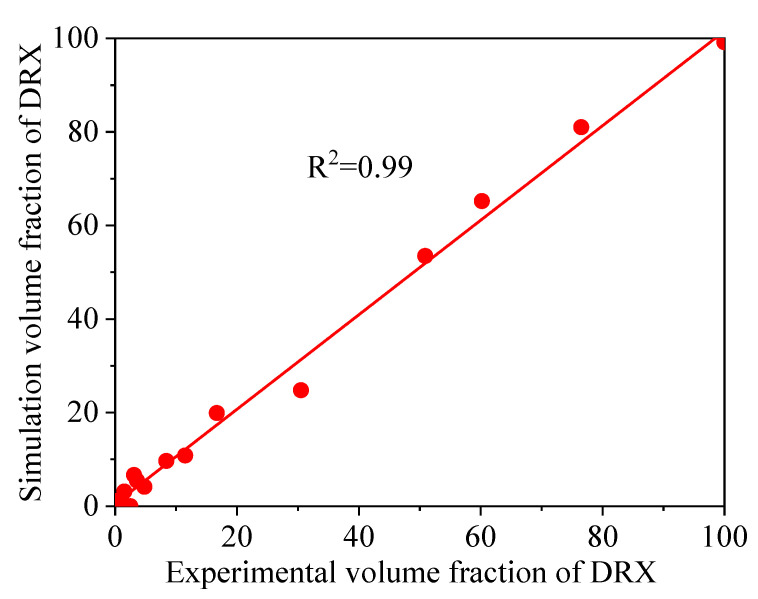
Correlation between the FEM and experimental volume fraction of DRX for 47Zr-45Ti-5Al-3V alloys after hot deformation.

Figure 11 and Figure 12 illustrate a difference between the experimental and FEM results of $d_{DRX}$ and $X_{DRX}$, respectively. A good consistency is observed between experimental and the simulation results. The change trends of $d_{DRX}$ and $X_{DRX}$ for the experimental and the simulation results are consistent. According to References [33,40], the correlation between the experimental and the simulation results was characterized by the correlation coefficient ($R^{2}$) and the average absolute relative error (∆) value. It is seen from Figure 13 and Figure 14 that the experimental results keep a linear relationship with the FEM results. The value of $R^{2}$ was 0.95 and 0.99 for the $d_{DRX}$ and $X_{DRX}$, respectively. It is generally believed that DRX does not occur in the area where the $X_{DRX}$ percentage is less than 5%; thus, the ∆ value of the $d_{DRX}$ and $X_{DRX}$ for DRX grains was computed to be, respectively, 15.7% and 8.78%. This suggests that the DRX behavior of 47Zr-45Ti-5Al-3V alloy can be described well by the established kinetic equations that are embedded in the DEFORM-3D software.

### 3.5. CA of DRX Behavior

The CA method is a mathematical algorithm used to describe the evolution of a complex system in discrete space-time. Usually, the CA model consists of four basic elements: cell space, neighbor type, boundary conditions and cell state. The cell orientation is set to a random number from 1 to 180, and the cell transformation rule is “Moore neighbor”. From the viewpoint of dislocation density, the plastic deformation of metals is attributed to dislocation slip and climbing. In the process of hot-working, the dislocation density in the matrix increases with the increase of strain, and the evolution of the microstructure during the hot deformation is always accompanied by the change of dislocation density. In the CA model, the dislocation model can be described as follows [22,41]:

(12) $$\sigma =\alpha \mu b\sqrt{\bar{\rho }}$$

(13) $$\frac{d\rho }{d\varepsilon }=k_{1}\sqrt{\rho }-k_{2}\sqrt{\rho }$$

where the α is 0.5; ρ and $\bar{\rho }$ represent the dislocation density and average dislocation density respectively; *b* represents the Burger’s vector; μ represents the shear modulus; and *k_1_* and *k_2_* represent work-hardening and dynamic-softening coefficients, respectively. There is no ρ gradient inside a single grain of alloy during the hot-working. The $\bar{\rho }$ during the CA simulation can be expressed as follows [42]:

(14) $$\bar{\rho }=\frac{1}{N_{0}}\sum_{i,j}^{i=A,j=B}\rho_{i,j}$$

where A and B respectively represent the number of cells in the *i* and *j* directions; *N_0_* represents the total number of cells; and $\rho_{i,j}$ represents the dislocation density of the cell at coordinates (*i*, *j*). During the hot-working process, as the dislocation density reaches the critical dislocation density ($\rho_{c}$), the DRX grains begin to nucleate. In addition, the formation of DRX grains reduces the ρ of the alloy, and a new round of DRX occurs as the amount of deformation further increases. The nucleation rate ($\dot{n}$) is linearly related to the $\dot{\varepsilon }$ [22,42]:

(15) $$\dot{n}=C{\dot{\varepsilon }}^{\alpha }$$

where *C* and α usually take 200 and 0.9, respectively.

**Figure 15:**
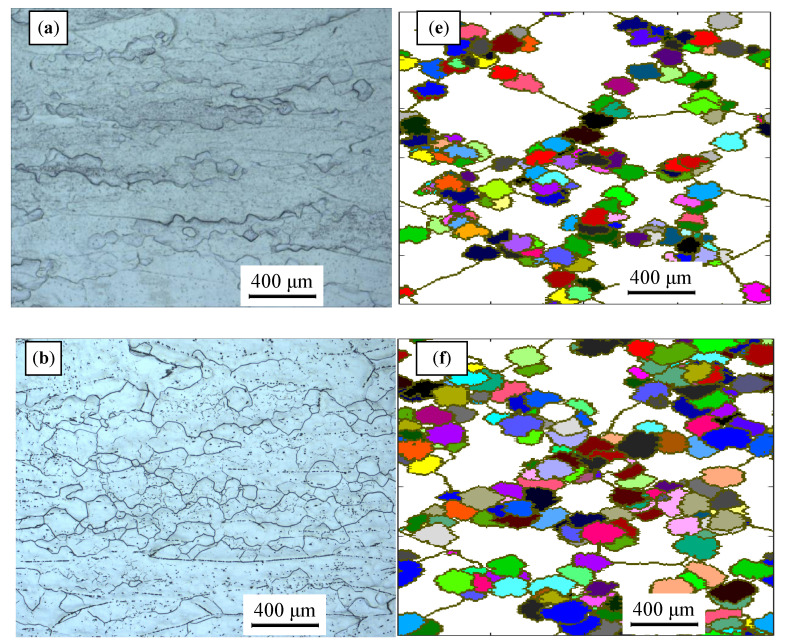

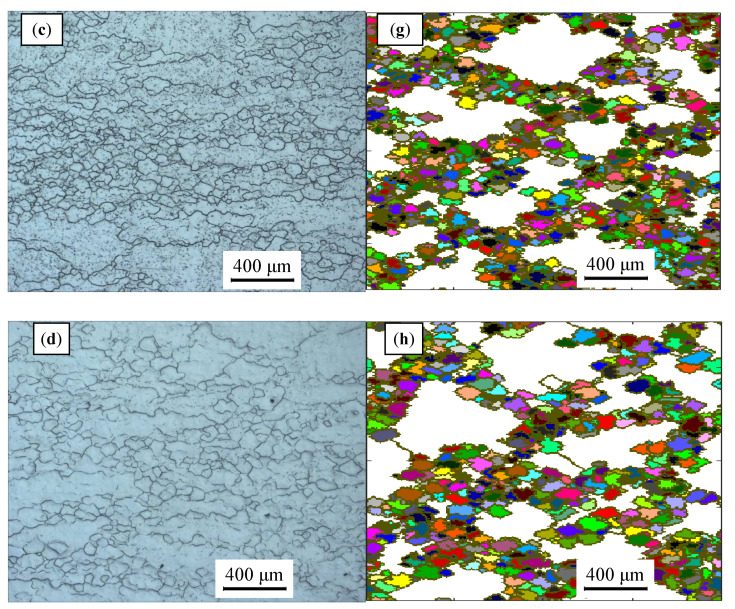
Microstructure of the 47Zr-45Ti-5Al-3V alloys after hot-working and CA simulation result: (**a**,**e**) 900 °C/10^−3^ s^−1^, (**b**,**f**) 950 °C/10^−3^ s^−1^, (**c**,**g**) 1000 °C/10^−2^ s^−1^ and (**d**,**h**) 1050 °C/10^−2^ s^−1^.

**Figure 16:**
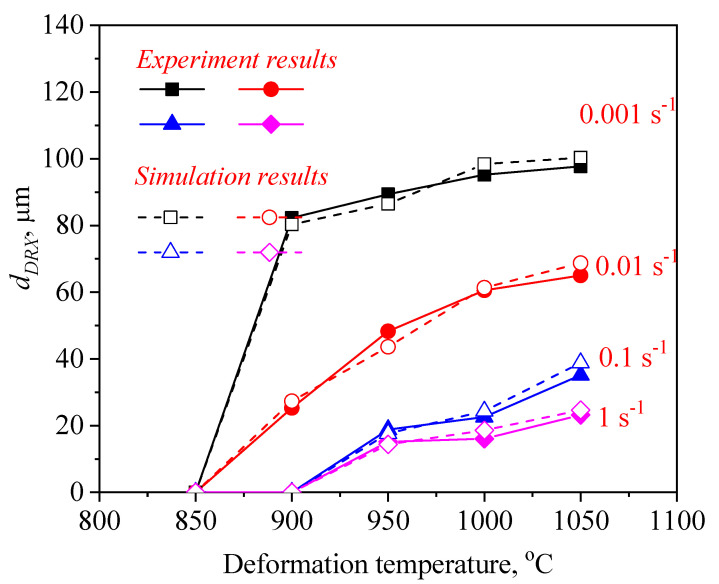
A comparison between the experimental and CA results of the $d_{DRX}$.

**Figure 17:**
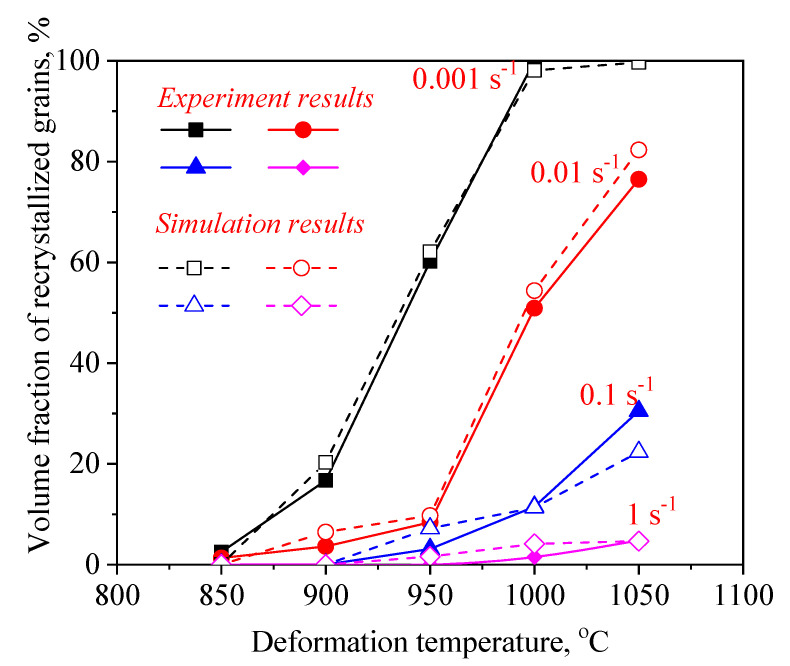
A comparison between the experimental and CA simulation results of the $X_{DRX}$.

Based on the initial microstructure of the solution-treated alloy, the initial solid solution structure was simulated by the CA simulation, as shown in Figure 1b. The experimental and predicted microstructures by CA after hot-working were displayed in Figure 15. The CA simulation results show that the evolution of DRX grains is strongly correlated with hot processing parameters. Figure 16 and Figure 17 reveal that the experimental results keep a linear relationship with the CA results. The ∆ value of the $d_{DRX}$ and $X_{DRX}$ for DRX grains was computed to be respectively 6.32% and 9.3%. In general, CA simulation has more accurate results than FEM simulation. However, FEM can more intuitively simulate the overall change of the alloy during the whole process of hot compression. The combination of FEM and CA simulation can more effectively predict the macro and micro evolution of DRX for 47Zr-45Ti-5Al-3V alloy.

## 4. Conclusions

The DRX behavior of the 47Zr-45Ti-5Al-3V alloy was investigated by using a CA–FE simulation. The main conclusion obtained can be drawn as follows:

(1) In this present study, the main softening mechanism of 47Zr-45Ti-5Al-3V alloy was regarded as DRX. The results revealed that the deformation *T* and $\dot{\varepsilon }$ have a strong effect on the DRX behavior of 47Zr-45Ti-5Al-3V alloy. The $X_{DRX}$ and $d_{DRX}$ of DRX grains increased with rising *T* and decreasing $\dot{\varepsilon }$.
(2) Based on the hot-working test, the $X_{DRX}$ and $d_{DRX}$ model of DRX were established, which can be written as the following formula:
  $$\left\{\begin{array}{c} X_{DRX}=1-exp\left[-0.0021\times {\left(\frac{\varepsilon -\varepsilon_{c}}{\varepsilon_{p}}\right)}^{1.88}\right]\\ d_{DRX}=2565.73Z^{-0.25} \end{array}\right\}$$
(3) The value of $R^{2}$ was, respectively, 0.95 and 0.99 for the $d_{DRX}$ and $X_{DRX}$ between the experimental and FEM results, while the average ∆ value for the $d_{DRX}$ and $X_{DRX}$ was, respectively, 15.7% and 8.78%, which indicated that the FEM results of $X_{DRX}$ and $d_{DRX}$ are in great line with the experimental results.
(4) The ∆ value of the $d_{DRX}$ and $X_{DRX}$ for DRX grains is respectively computed in the process of CA simulation. The ∆ value of the $d_{DRX}$ and $X_{DRX}$ was, respectively, 6.32% and 9.3% for CA simulation, which indicated that CA simulation has more accurate results than FEM.

## Figures and Tables

**Table 1:** The values of $\varepsilon_{p}$ and $\varepsilon_{c}$ under various hot-working conditions.

Temperature	$\varepsilon_{p}$				$\varepsilon_{c}$			
	1 s^−1^	0.1 s^−1^	0.01 s^−1^	0.001 s^−1^	1 s^−1^	0.1 s^−1^	0.01 s^−1^	0.001 s^−1^
850 °C	0.38	0.21	0.12	0.07	-	-	-	-
900 °C	0.34	0.14	0.11	0.055	-	-	0.07	0.038
950 °C	0.26	0.1	0.083	0.025	0.17	0.06	0.05	0.018
1000 °C	0.17	0.08	0.03	0.012	0.13	0.05	0.018	0.006
1050 °C	0.13	0.05	0.02	0.01	0.09	0.03	0.01	0.004

**Table 2:** The distribution of $d_{DRX}$ for 47Zr-45Ti-5Al-3V alloys deformed under various hot-working conditions at the true strain of 0.7.

Strain Rate/s^−1^	Temperature/°C				$d_{DRX},\;\mu m$
	900	950	1000	1050	
0.001	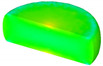	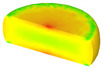	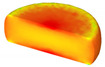	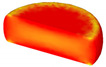	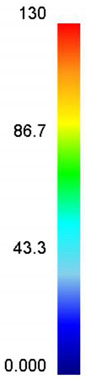
0.01	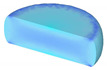	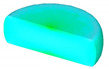	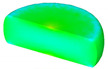	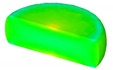	
0.1	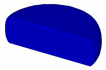	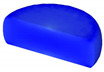	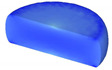	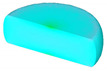	
1	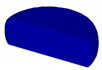	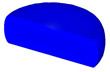	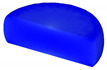	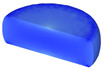	

**Table 3:** The distribution of $X_{DRX}$ for 47Zr-45Ti-5Al-3V alloys deformed under various hot-working conditions at the true strain of 0.7.

Strain Rate/s^−1^	Temperature/°C				$X_{DRX},\%$
	900	950	1000	1050	
0.001	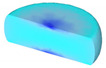	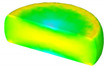	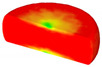	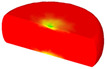	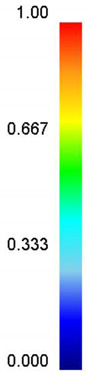
0.01	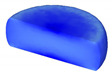	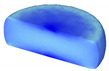	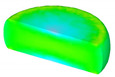	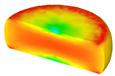	
0.1	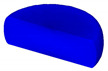	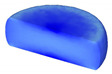	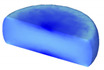	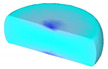	
1	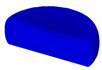	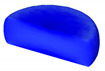	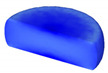	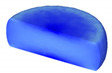	

## Data Availability

Data sharing is not applicable to this article.
